# Neurotrauma Registries in Low- and Middle-Income Countries for Building Organized Neurotrauma Care: The LATINO Registry Experience: Comment on "Neurotrauma Surveillance in National Registries of Low- and Middle-Income Countries: A Scoping Review and Comparative Analysis of Data Dictionaries"

**DOI:** 10.34172/ijhpm.2022.7505

**Published:** 2022-08-24

**Authors:** Andrés M. Rubiano, Angelica Clavijo

**Affiliations:** ^1^Universidad El Bosque, Bogotá, Colombia.; ^2^Meditech Foundation, Cali, Colombia.

**Keywords:** LMICs, Neurotrauma, Trauma Registry, Trauma Systems and Latin-America

## Abstract

Trauma registries play an important role in building capacity for trauma systems. Regularly, trauma registries exist in high-income countries (HICs) but not in low- and middle-income countries (LMICs). Neurotrauma includes common conditions, like traumatic brain injuries (TBIs) and spinal cord injuries. The development of organized neurotrauma care is crucial for improving the quality of care in less-resourced areas. The recent article published in *International Journal of Health Policy and Management* by Barthélemy et al entitled "Neurotrauma Surveillance in National Registries of Low- and Middle-Income Countries: A Scoping Review and Comparative Analysis of Data Dictionaries" adds an important body of literature to improve understanding of the importance of these types of efforts by promoting organized neurotrauma care systems in LMICs. Here, we provide a short commentary based on our experience with the Latin America and the Caribbean Neurotrauma Registry (LATINO-TBI) in the Latin America (LATAM) region.

## Introduction

 Trauma registries are fundamental in the development of organized trauma and emergency care systems globally. They help to support a wide range of processes, including quality improvement and policy development.^1,2^ They also support research efforts and allow the comprehension of the disease from both epidemiological and financial perspectives.^3,4^ In the current study by Barthélemy et al,^5^ it is important to understand that neurotrauma registries in low- and middle-income countries (LMICs) are heterogeneous with limitations in the use of common data elements, generating a barrier for comparisons between studies and for the development of further meta-analyses.

 Other difficulties that have been found in registry implementation efforts of LMICs include low data quality and barriers generated by administrators who do not consider this type of effort a priority for local funding.^6^ We recognize from firsthand experience many of these issues, from the development of the Latin America and the Caribbean Neurotrauma Registry (LATINO-TBI). We present a brief commentary regarding this experience and how we faced the same barriers discussed in the publication of Barthélemy et al.

## The LATINO Neurotrauma Registry

 The LATINO neurotrauma registry is a data collection instrument based on the National Institutes of Health (NIH) common data elements for traumatic brain injury (TBI) and adapted to the World Health Organization (WHO) recommendations for the basic elements for global trauma registries. The combination of these perspectives allows this effort to be adaptable to different contexts, like the ones that we found in the Latin America (LATAM) region.^7^

 In this registry, even centers with fewer resources can participate and transition from a minimal data set to a more complete one as they increase the level of development and support from local administrative authorities. Additionally, to overcome language barriers, the LATINO-TBI registry is available in Spanish, English, and Portuguese, which are the most common languages in the region, with the same internal coding to facilitate cross-country analysis and comparative analysis with other databases.

 The registry project is available at https://www.latinotbi.com/. It is an open project that allows centers in the region to analyze their data individually and participate in multi-institutional analysis. Mostly, we face barriers associated with the lack of local administrative support at participating institutions and incomplete data, especially in sections like prehospital care and outcome data after hospital discharge.

## Organized Neurotrauma Care and the Burden of the Disease

 Organized neurotrauma care has been associated with improved outcomes and decreased burden of the disease. The concept of organized neurotrauma care includes aspects from prevention to rehabilitation services, crossing echelons of care at prehospital, emergency department, surgery, and intensive care levels The neurotrauma registries allow for the evaluation of interventional impact at all these stages. Systemic data, including demographic and epidemiological variables, urgent care variables, and critical and chronic care variables allow us to understand the ecology of care, and evaluate critical aspects like cost and effectiveness of interventions.^8,9^

 In LMICs, these data are difficult to find in past and present studies. Efforts like the study of Barthélemy et al, pooling data from the few available studies, show the flaws and barriers present in the actual literature and support the requirement of better data collection systems for neurotrauma studies in these areas globally.^10^

**Figure 1:**
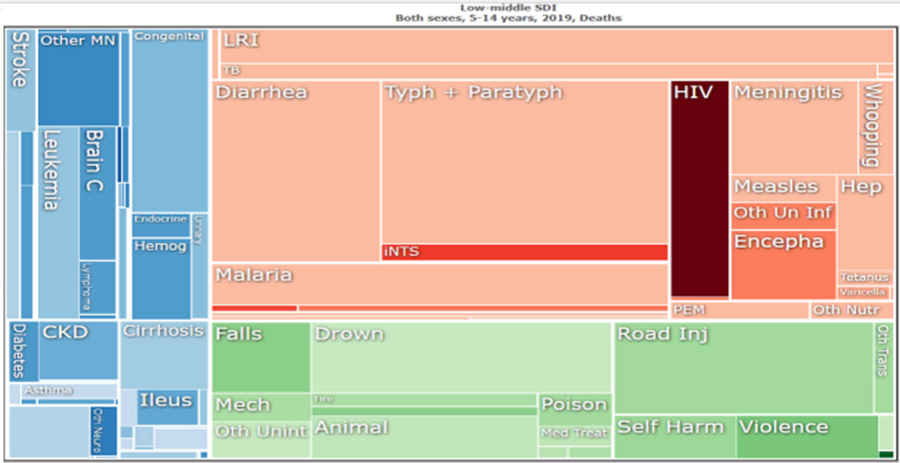
Impact of Trauma Burden (Green Boxes) in the Mortality of the 5-14 Year-Old Population (Both Sexes) of LMICs, According to the Low-Middle HSDI. Data are based on the global burden of diseases study (GBD Study) from the Institute for Health Metrics and Evaluation. Graphs made using GBD software. Abbreviations: HSDI, Human Social Developing Index; LMICs, low- and middle-income countries.

**Figure 2:**
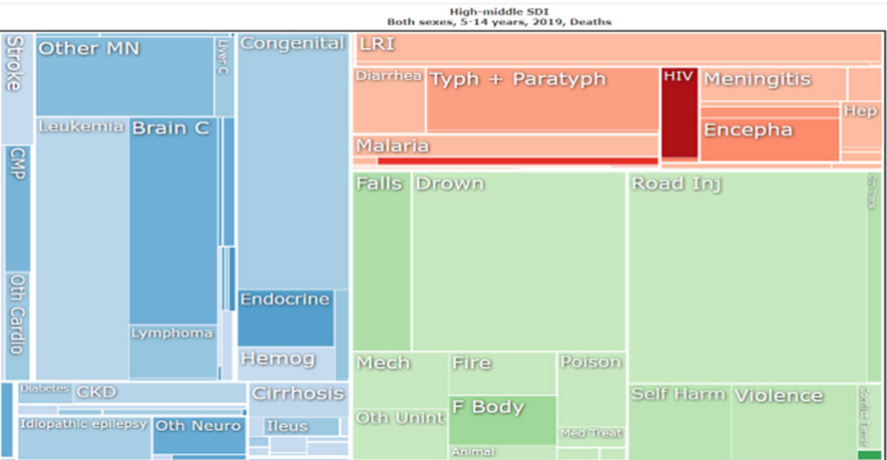
Impact of Trauma Burden (Green Boxes) in the Mortality of the 5-14 Year-Old Population (Both Sexes) of HICs, According to the High-Middle HSDI. Data are based on the global burden of diseases study (GBD Study) from the Institute for Health Metrics and Evaluation. Graphs made using GBD software. Abbreviations: HSDI, Human Social Developing Index; HICs, high-income countries.

 TBI is considered the most common condition requiring medical and surgical management worldwide, affecting both high-income countries (HICs) and LMICs. Neurotrauma is a disease prevalent in areas that are more dependent on personal transportation (non-public transport), especially motorcycles. Motorcycles are the preferred vehicles in LMICs, generating more risk due to low road quality, poor preventive policies, and low level of reinforcement of healthy behaviors when driving.^11,12^ Differences in metrics of the Global Burden of Diseases Study show that the burden of trauma affects mostly the young population globally (Figure 1). The data in LMICs — and especially in low-income countries — is misrepresented due to the low availability of sound data registries associated with trauma care (Figure 2).^13^ These issues have been recently discussed in papers published by the group of investigators from the Global Surgery and Social Change of Harvard University,^14,15^ where important figures from the global neurosurgery movement have been trained, including the author of the commented *International Journal of Health Policy and Management* paper, Dr. Barthélemy.

## Conclusion

 Neurotrauma registries are fundamental for capacity building in organized neurotrauma care. Heterogeneity is present in some registries, but especially in registries in LMICs. Barriers include lack of support from local hospital administrators and difficulties in obtaining prehospital and outcome data after discharge. We need harmonization of neurotrauma registries with efforts like the NIH common data elements for TBI. The LATINO neurotrauma registry is an example of a neurotrauma registry based in LMICs that fills gaps in TBI management within LMICs.

## Ethical issues

 Not applicable.

## Competing interests

 Authors declare that they have no competing interests.

## Authors’ contributions

 AMR and AC develop the idea, write and checked the final version of the manuscript.

## References

[1] Mowafi H, Ngaruiya C, O’Reilly G (2019). Emergency care surveillance and emergency care registries in low-income and middle-income countries: conceptual challenges and future directions for research. BMJ Glob Health.

[2] Lecky FE, Reynolds T, Otesile O (2020). Harnessing inter-disciplinary collaboration to improve emergency care in low-and middle-income countries (LMICs): results of research prioritisation setting exercise. BMC Emerg Med.

[3] Mobinizadeh M, Berenjian F, Mohamadi E (2022). Trauma registry data as a policy-making tool: a systematic review on the research dimensions. Bull Emerg Trauma.

[4] Rosenkrantz L, Schuurman N, Arenas C, Nicol A, Hameed MS (2020). Maximizing the potential of trauma registries in low-income and middle-income countries. Trauma Surg Acute Care Open.

[5] Barthélemy EJ, Hackenberg AEC, Lepard J (2022). Neurotrauma surveillance in national registries of low-and middle-income countries: a scoping review and comparative analysis of data dictionaries. Int J Health Policy Manag.

[6] Dasari M, Johnson ED, Montenegro JH (2021). A consensus statement for trauma surgery capacity building in Latin America. World J Emerg Surg.

[7] Johnson ED, Oak S, Griswold DP, Olaya S, Puyana JC, Rubiano AM (2021). Neurotrauma registry implementation in Colombia: a qualitative assessment. J Neurosci Rural Pract.

[8] Rolle ML, Garba DL, Rubiano AM (2021). Commentary: the need for a Global Neurotrauma Registry in Caribbean nations. Neurosurgery.

[9] Bommakanti K, Feldhaus I, Motwani G, Dicker RA, Juillard C (2018). Trauma registry implementation in low-and middle-income countries: challenges and opportunities. J Surg Res.

[10] Smith BG, Whiffin CJ, Esene IN (2021). Neurotrauma clinicians’ perspectives on the contextual challenges associated with long-term follow-up following traumatic brain injury in low-income and middle-income countries: a qualitative study protocol. BMJ Open.

[11] Fernández Londoño LL, Marchesini N, Espejo Ballesteros D (2022). Epidemiological review of spinal cord injury due to road traffic accidents in Latin America. Med PrincPract.

[12] Dunne J, Quiñones-Ossa GA, Still EG (2020). The epidemiology of traumatic brain injury due to traffic accidents in Latin America: a narrative review. J Neurosci Rural Pract.

[13] James SL, Theadom A, Ellenbogen RG (2019). Global, regional, and national burden of traumatic brain injury and spinal cord injury, 1990-2016: a systematic analysis for the Global Burden of Disease Study 2016. Lancet Neurol.

[14] Dewan MC, Rattani A, Gupta S, et al. Estimating the global incidence of traumatic brain injury. J Neurosurg. 2018:1-18. 10.3171/2017.10.jns17352. 29701556

[15] Kumar R, Lim J, Mekary RA (2018). Traumatic spinal injury: global epidemiology and worldwide volume. World Neurosurg.

